# Dispersal patterns of an introduced wild bee, *Megachile sculpturalis* Smith, 1853 (Hymenoptera: Megachilidae) in European alpine countries

**DOI:** 10.1371/journal.pone.0236042

**Published:** 2020-07-10

**Authors:** Julia Lanner, Katharina Huchler, Bärbel Pachinger, Claudio Sedivy, Harald Meimberg

**Affiliations:** 1 Institute for Integrative Nature Conservation Research, University of Natural Resources and Life Sciences Vienna (BOKU), Vienna, Austria; 2 Wildbiene + Partner AG, Zürich, Austria; National Taiwan Normal University, TAIWAN

## Abstract

Biodiversity monitoring programs are the baseline of species abundancy studies, which in case of introduced species are especially critical. *Megachile sculpturalis* Smith, 1853 native to Eastern-Asia, constitutes the first ever recorded wild bee species accidently introduced in Europe. Since its first discovery in 2008, *M*. *sculpturalis* has been spreading across the continent. By initiating a citizen science monitoring program, we aimed to investigate the occurrence pattern of *M*. *sculpturalis*. Within only two years after starting the project, 111 new reports from Switzerland, Liechtenstein and Austria were recorded. Comparably to other European countries, the population progressed remarkably fast from year to year expanding its area geographically but also ecologically by increasing its altitudinal range. The distribution pattern indicates human assisted jump-dispersal travelling on the major traffic routes of central Europe.

## Introduction

Citizen science is a century-old practice, wherein research projects are carried out in the public by including volunteers. Scientists together with non-professionals collect, analyze or interpret various data sets [1]. By including citizens into surveillance monitoring programs, ecological research questions can be approached on a broader scale, both temporally and spatially, on private land and with less expense [2–4]. Those benefits contribute to the popularity of citizen science projects (CSP) reflected by countless research topics published within the last years [5–7]. Although insects in general potentially evoke negative perceptions in humans [8,9], pollinators like bumble bees and butterflies are attractive groups for CSP [10–15]. Most CSP focus on creating data sets of species distribution records [3], which constitute the baseline of abundance studies [16–19].

Species distribution studies are of great interest in environmental research and conservation planning, and one of their multiple applications is to monitor species outside their original home range or introduced taxa that become invasive [20]. As invasive species constitute one of the main threats to a declining entomofauna and biomass [21–23], gathering information about their population progression is crucial. Previous CSP have proved to be hugely beneficial in detecting introduced communities, their range expansions and negative interspecific interactions on population declines of native species [24,25].

In 2018, approximately 40 introduced bee species were reported in North America [26]. *Megachile sculpturalis* Smith, 1853 is one of these non-native wild bees in North America andnative to Asia (China, Japan, Tibet, Korean Peninsula and East Russia) [27–29]. It was first observed in 1994 at the Campus of the North Carolina State University [30] and throughout the next two centuries, *Megachile sculpturalis* expanded its distribution in the US successfully [31–33]. In comparison to North America, European mainland was untouched by introduced wild bees for a long period of time [34–37]. But in 2008, *M*. *sculpturalis* was found at a European country in Aullach (France) and became the first recorded wild bee accidently introduced on the continent [38]. *Megachile sculpturalis* most likely arrived in the little city near Marseille by shipping, spreading along the major maritime trading routes as a stowaway [38,39]. In the following year, *M*. *sculpturalis* was reported from northern Italy at the Lake Maggiore area [40] and within the next years also north of the alps in Switzerland and South Germany [41–43]. In the following years, single observations of *M*. *sculpturalis* were reported from the Central European countries Austria [44], Slovenia [45], Hungary [46] and from the Crimean peninsula [47]. In 2013, *M*. *sculpturalis* was observed near the Catalonian border in Matemale (France) at a new maximum of 1540 m above sea level [42]. *Megachile sculpturalis* was found in several locations in Catalonia in 2018 [48]. In the meantime, *M*. *sculpturalis* expanded its range in France colonizing 72 French cities with the highest population density recognized in southern areas around the origin of its first discovery, Marseille. Alpine mountain chains seem to be no limiting factor for its dispersal in France. Nevertheless, observations were missing for some French areas, for example eastern regions (Côte d’Or and Jura) near the border to Switzerland [39].

However, it remained unclear if gaps in the species documented biogeography in countries like Switzerland and Austria indeed reflected distribution gaps or rather resulted from inquiry gaps. But in contrast to other species from the Anthophila clade, the identification of *M*. *sculpturalis* does not require taxonomic skills from experts [38,49]. The species is characterized by its outstandingly large body size of 17–22 mm for males and 21–27 mm for females, its infuscated wings and its bright orange hairy thorax which contrasts with the narrow body [32,33,41]. Additionally, *M*. *sculpturalis* is a cavity-nesting species. As they are incapable of excavating their own nesting burrows, females use pre-existing holes in dead wood or reed with diameters between 8 and 20 mm and frequently occupy artificial nests (“bee hotels”), where they can be easily observed [39,50]. While the partition walls between the egg chambers are covered by a mud layer, the lateral walls and the entrance of the cavities are sealed with resin [33,42]. By examining the resin plug at the nest entrances of *M*. *sculpturalis*, the occupied cavities can be distinguished reliably from those of mason or carpenter bees. These characteristics facilitate identification and make the species suitable for a CS program.

Here, we present species distribution records of three alpine countries of the first accidentally introduced wild bee on European mainland, *M*. *sculpturalis*. We aimed to collect records in Austria, Liechtenstein and Switzerland by an international citizen science program started in 2018. In these three countries we assumed to find an already established population of *M*. *sculpturalis*, although published records were scarce. Investigating the spatial and altitudinal distribution of records we determined an exceptional fast range expansion over long distances. Using different estimates for the species range we discuss different dispersal mechanisms within the alpine region.

## Material and methods

### Study organism

**Fig 1:**
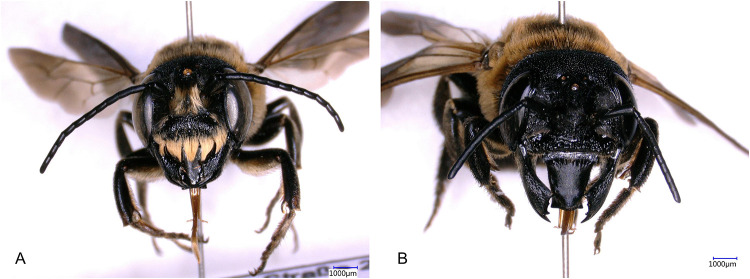
Sexual dimorphism of A: male and B: female of the introduced wild bee *Megachile sculpturalis*. (2-column fitting image).

*Megachile sculpturalis* is the only species of the subgenus *Callomegachile* Michener, 1962 in Europe [41]. Due to its unique appearance, species identification and gender differentiation (males show a bright hairy supraclypeal plate and females with ventral scopae) was accomplished in the field and through pictures (Fig 1). It is a protandrous species, where males emerge earlier than females. Their flying season starts in late June to early July and generally ends in in mid-September [40,51]. It is a polylectic species feeding on many different plant taxa. The majority of the wild bee’s host plants in North America are of Asian origin, where they were observed collecting pollen on *Ligustrum lucidum* (Oleaceae) or *Buddleia spp*. (Loganiaceae) [30,33]. A similar picture is presented in Europe as *M*. *sculpturalis* tends to collect nectar on many different flowers and pollen with a high preference for *Ligustrum sp*. and *Styphnolobium japonica* [39,48,50,52].

### Data collection

In accordance to Miller-Rushing *et al*. [1] and Shirk *et al*. [111] data from Austria and Switzerland was collected by a classical contributory citizen science study design, where mainly non-experts collect observations working together with experts, who verify the data. Several international calls for reports were published with the support of companies, nature platforms, nature museums and entomological clubs (S1 Table). Volunteers were asked to send their observations of *M*. *sculpturalis* via email to: asiatische_moertelbiene@outlook.com. Most participants were amateurs with a strong interest in insects, but a few professionals contributed too. Observations were asserted as verified with a valid photo, video and dead or living specimens in order to exclude errors and misidentifications from the data set. Starting the CSP in Switzerland and Austria in spring in 2018, occurrence reports presented here were recorded until December 2019.

We asked when the observation was made and if the bee has been observed in the previous years at the same site. With this procedure the absence of the species before a certain year had been recorded. Such backdated observations were incorporated as well, thus, we retrospectively generated data to years before the CSP started. In addition, years before the report without any observation at the same time might indicate absences. Participants were asked to describe observations concerning nesting activity, plant interactions and other peculiarities to collect information about ecological requirements of the species. When the bee was recognized during nesting, participants were asked to describe and to provide images of the nesting site, if possible (e.g. artificial nest, wood furniture, wooden cavity build by insects or others, examples are shown in S2 and S3 Tables). Furthermore, participants were asked to observe the species during its active season and report agonistic behavior. An additional question was, how they became aware of the described citizen science project, for future reference.

### Spatial estimations

All records were included in a table indicating, the year, zipcode, coordinates and observations (Table 1, S2 and S3 Tables). Data were analyzed according reports, number of records, and validated records. Observations of more than one individual per year were not considered to increase comparability between participants. Additionally, we incorporated 34 observations of *M*. *sculpturalis* reported in the literature [41–45,52–55] and 10 reports from public platforms, such as iNaturalist (© California Academy of Sciences 2016) and info fauna (Schweizerisches Zentrum für die Kartografie der Fauna SZKF/CSCF, Neuchâtel 2010), which led to a total number of 155 analyzed records.

**Table 1:** Occurrence data of *Megachile sculpturalis* gathered by citizen scientists in Austria, Liechtenstein and Switzerland from the year 2016 until 2019.

Country	Location	Year of observation	coordinates N	coordinates E	altitude (m.a.s.l.)
Austria	Bregenz	2019	47,504194	9,758872	407
	Bregenz	2019	47,497922	9,718153	412
	Bregenz	2019	47,49637	9,708246	472
	Dornbirn	2019	47,430903	9,731998	411
	Eisenstadt	2019	47,8501	16,519527	229
	Frastanz	2019	47,219921	9,625075	489
	Grän	2019	47,495519	10,556111	1111
	Graz	2019	47,072756	15,435012	347
	Hirschegg	2019	47,351331	10,148043	1230
	Höchst	2019	47,461482	9,625114	402
	Hohenems	2019	47,363619	9,682706	428
	Hohenweiler	2019	47,58614	9,779308	496
	Hörbranz	2019	47,55528	9,752778	428
	Klagenfurt	2019	46,616461	14,277077	443
	Lauterach	2019	47,474613	9,729087	414
	Lauterach	2019	47,726546	9,60134	414
	Lauterach	2019	47,479776	9,736774	414
	Lingenau	2019	47,445328	9,921635	680
	Lustenau	2019	47,4409	9,654052	403
	Lustenau	2019	47,447661	9,664043	405
	Lustenau	2018, 2019	47,440514	9,650272	405
	Lustenau	2019	47,440459	9,65087	406
	Lustenau	2019	47,426229	9,687023	405
	Meiningen	2019	47,29	9,58	429
	Radfeld	2019	47,452253	11,91229	514
	Salzburg	2019	47,810463	13,038334	422
	Salzburg	2019	47,789872	12,987455	439
	Schruns	2019	47,077625	9,920829	683
	Wattens	2019	47,289536	11,598711	566
Switzerland	Affoltern am Albis	2018	47,279225	8,455325	499
	Andelfingen	2017	47,592684	8,675108	412
	Bern	2017	46,95293	7,397099	560
	Bern	2018	46,957463	7,450772	561
	Binz	2018	47,357319	8,626778	630
	Chavannes-près-Renens	2018	46,536636	6,578023	419
	Crassier	2017	46,374331	6,164548	475
	Emmen	2018	47,078331	8,266719	498
	Emmetten	2016	46,95618	8,510671	760
	Endingen	2019	47,537143	8,294401	428
	Feldmeilen	2019	47,27536	8,624631	424
	Frauenfeld	2019	47,55354	8,8819	407
	Frauenfeld	2019	47,554114	8,912028	412
	Frauenfeld	2018, 2019	47,553384	8,893356	471
	Fürstenau	2017	46,7209	9,446807	664
	Genf	2018	46,181846	6,141118	387
	Genf	2018	46,447231	6,160412	1048
	Gerra/Tessin	2018, 2019	46,122088	8,784991	217
	Glattpark	2017	47,422164	8,562512	424
	Hemmiken	2018	47,48802	7,891359	501
	Höngg, Zürich	2018	47,40166	8,497715	398
	Hunzenswil	2018	47,387444	8,124859	660
	Konolfingen	2017, 2019	46,876123	7,622538	549
	Langnau a.A.	2017	47,284773	8,531174	488
	Laupen	2018, 2019	46,907115	7,23727	463
	Lausanne	2018, 2019	46,533418	6,621065	583
	Lausanne	2018, 2019	46,519135	6,621373	458
	Meggen	2018	47,04	8,37	452
	Meilen	2019	47,269286	8,652071	437
	Meilen	2019	47,270896	8,64464	447
	Neggio	2018	45,989187	8,882886	590
	Neuenegg	2018	46,89	7,3	608
	Oberhofen am Thunersee	2018, 2019	46,730836	7,673244	442
	Oberrieden	2017, 2018, 2019	47,281031	8,576063	413
	Rapperswil	2018, 2019	47,226977	8,821341	420
	Rickenbach Sulz	2018, 2019	47,551173	8,795435	421
	Rohrschach	2019	47,476869	9,482317	229
	San Nazzaro	2017	46,132615	8,805229	404
	Sankt Margarethen	2018, 2019	47,451277	9,643941	427
	Sankt Margarethen	2018	47,450532	9,630142	490
	Sargans	2018, 2019	47,048264	9,4402	490
	Sargans	2018	47,051305	9,430751	401
	Schlieren	2018	47,395096	8,442993	538
	Sierre	2018	46,292252	7,532319	476
	St-Blaise	2018, 2019	47,017665	6,991284	490
	Stäfa	2017	47,248969	8,722076	579
	Steffisburg	2017	46,769112	7,631555	416
	Stein am Rhein	2018	47,655819	8,857956	501
	Tartegnin	2016	46,466655	6,31568	603
	Trimmis	2017	46,901023	9,562869	731
	Unterlangenegg	2019	46,790869	7,686887	428
	Unterlunkhofen	2017	47,323509	8,38259	648
	Visp	2018, 2019	46,292884	7,886431	395
	Wettingen	2017	47,459379	8,319725	540
	Wetzikon	2016	47,332938	8,786572	599
	Wilen	2017, 2019	46,87933	8,215595	436
	Zürich	2018	47,426885	8,545503	402
	Zürich	2018	47,40924	8,54477	477
	Zürich	2018	47,36966	8,55319	433
	Zürich	2019	47,404915	8,485528	450
	Zürich	2019	47,372394	8,542333	451
	Zürich	2019	47,361573	8,529741	412
	Zürich	2017	47,41	8,54	452
	Zürich	2019	47,380806	8,501681	444
	Zürich	2018	47,391144	8,541656	446
Liechtenstein	Balzers	2019	4.706.312	949.169	478

Spatial analysis was conducted using ArcGIS 10.6 [56] and R 3.6.1 [57]. We calculated each points elevation (given in m ASL) from a digital elevation model provided by the Copernicus Program [58] and descriptively investigated minimum, maximum and mean (± standard deviation) elevation per country.

To estimate the potential distribution range and roughly assess range expansion within the alpine region, we applied two simple approaches that do not require absence data or further assumptions on ecological requirements [59–61]. The first approach investigates the extent of occurrence of a species, for example for IUCN Red List assessments, by calculating the minimum convex polygon. This is the smallest possible polygon with no internal angle greater than 180° containing all data points [61,62]. We computed this minimum convex polygon by applying the Minimum Bounding Geometry tool of ArcGIS (later referred to as ‘approach D’). However, the minimum convex polygon does not account for distribution ranges that include discontinuities and thus is frequently replaced by calculating the α-convex hull [59,62–65]. The α-convex hull is a generalization of the convex hull and allows for enclosed gaps and disconnected shapes due to its relaxed assumptions of connectivity [59,63]. We calculated the α-convex hull based on Voronoi diagrams and Delaunay triangulations [60]. The α-convex hull is ultimately defined by the parameter α, which affects the entire shape of the output [60]. Alpha was chosen in advance and set to three different values reflecting a varying extent of stringency according to the literature (for example [64,66–75]. The most conservative approach (later referred to as ‘approach A’) in choosing α, encompassing the least area, was calculated with α = 0.8, as this value was at the verge of producing complete hulls rather than a mesh between observations. For the radical approach (later referred to as ‘approach C’) we set α = 1.8, because it was the smallest value resulting in one shared hull approximately including all data points to avoid a disjunct distribution and, consequentially, encompassed the largest area of the α convex hull reconstruction [64,67,69]. As intermediate approach (later referred to as ‘approach B’), we chose an average value of α = 1.3. We calculated the α-convex hulls using the R package *‘alphahull’* [60] and processed the resulting data with the packages *‘sf’* [76] and *‘sp’* [77]. Resulting shapefiles were imported into ArcGIS to calculate the area that was enveloped by each hull.

All maps were depicted using the projected coordinate system ETRS89 LAEA Europe and several open data sources. The digital elevation model, as well as all data on standing water bodies was obtained from EU-DEM v1.1 and EU-Hydro River Network Database of the Copernicus Program [78,79]. Moving water bodies were gathered from the Rivers and Lake Centerlines data set as well as the corresponding Europe supplement of the Natural Earth program in large scale resolution of 1:10m [80]. Administrative borders were received from EuroGraphics [81]. Furthermore, data provided by the Swiss Federal Office of Topography “Swisstopo” on Swiss cantons (states) were used for localizing the administrative position of data points [82]. On all maps, data points were indicated by their data source (CSP vs. public platforms and published literature) and, if necessary, observation year (before 2016, 2016, 2017, 2018, 2019).

## Results

### Confirmed records and unverified observations

The CSP obtained 168 records, of which 111 (66%) could be affirmed. Of these verified records, 96 were within the year of report and 15 from the year before. 57 observations could not be confirmed: 13 (8%) were excluded either due to missing pictures or pictures without appropriate quality for an ensured identification of *M*. *sculpturalis*. Another 37 (22%) observations were unconfirmed as the reports comprised Hymenopterans but no *M*. *sculpturalis*: 8 observations of *Apis mellifera*, 5 *Xylocopa sp*., 3 *Megachile willughbiella*, and single observations showing *Andrena sp*, *Anthidium manicatum*, *Megachile parietina and Bombus hortorum*. Furthermore, nests of an unidentified *Bombus sp*. and *Osmia cornuta* were reported. Of the remaining 7 (4%) observations, the pictures depicted Syrphidae; 2 *Sceliphron curvatum*, one *Vespa crabro* and 5 unidentified wasp nests.

### Citizen science in Austria

For Austria, we received 30 verified records of *M*. *sculpturalis* from 20 different cities and six federal states (S2 Table). All 30 reports were received in 2019. Only one of the reports considered a nesting population observed in 2018, leaving 29 observations (= 100%) for descriptive analysis of supplementary information. In four cities (Bregenz, Lauterach, Lustenau, Salzburg), *M*. *sculpturalis* was observed multiple times in different localities. Reports of *M*. *sculpturalis* accumulated in the western parts of Austria. The majority of reports of *M*. *sculpturalis* from the western part of Austria were made close to the Swiss border. The observations were made at elevations from 229 m ASL in Eisenstadt up to 1230 m ASL in a western Austrian valley (Kleinwalsertal). The average elevation was 490.3 ± 204.3 m ASL. In northern regions (the federal states Upper and Lower Austria) only single observations were made.

Most data were obtained with the support of the nature museum “inatura” (76%). After the first two observations, news agencies were contacted in cooperation with “inatura”. Subsequently, local and national newspapers along with online news channels published articles of the project, which increased the likelihood of incoming reports. Another 10% of all observations in Austria were received with the support of the nature platform “naturbeobachtung”. The remaining reports were acquired by web research, social media (Instagram) and from one volunteer who directly contacted the institute.

**Fig 2:**
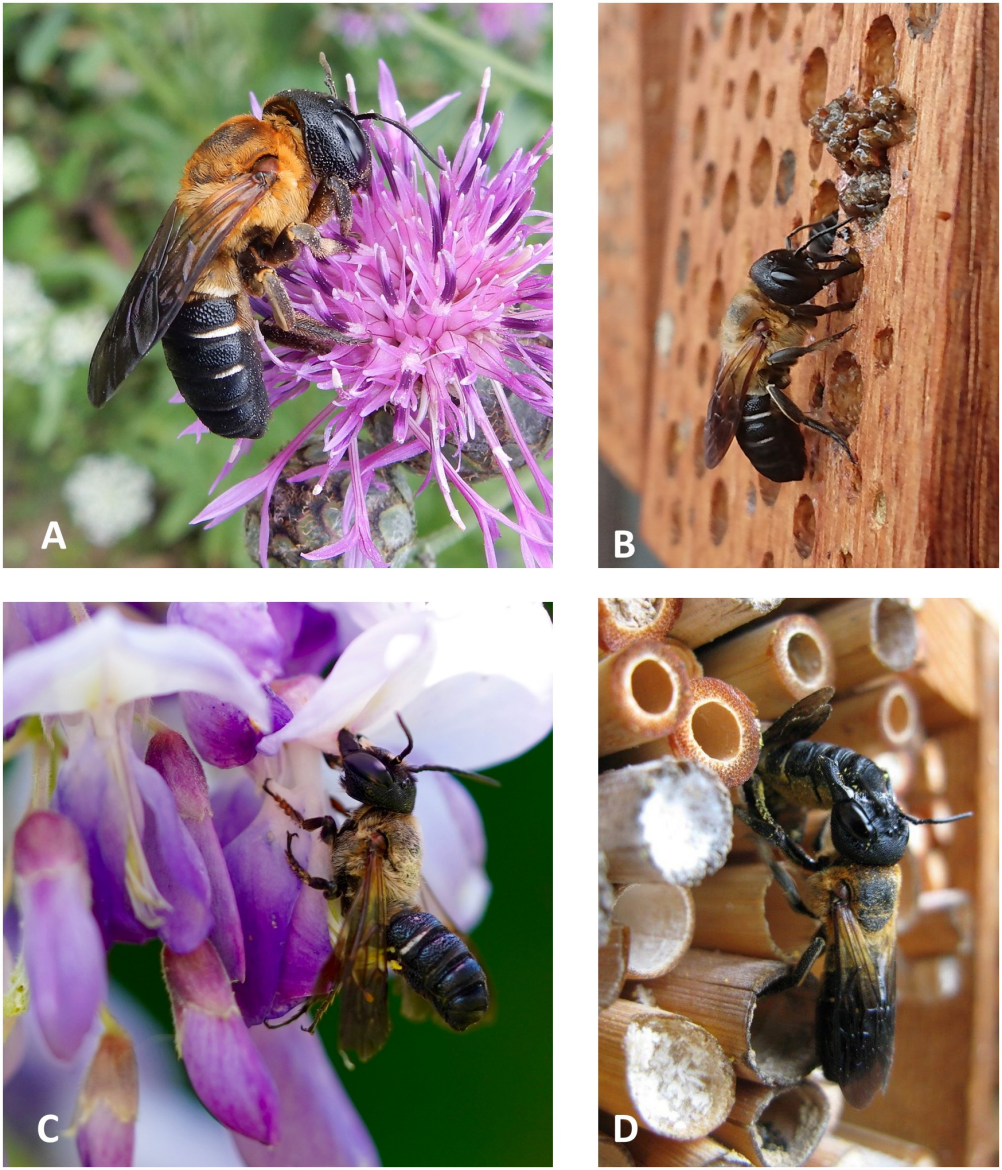
Participants were asked to send images or videos of their observations. Afterwards, the documentations were verified, and the volunteers got direct feedback on their observations. A: Female of *Megachile sculpturalis* found in Eisenstadt (Austria) at *Centaurea scabiosa*; B: In Salzburg (Austria), the species was observed nesting; C: A male bites into a blossom of *Wisteria* sp. in Lauterach (Austria); D: Two females show intraspecific competition for the biggest cavities of a trap nest (Oberhofen am Thunersee, Switzerland). Reprinted under a CC BY license, with permission from Hoffmann, Topitz, Seiwald and Merz, original copyright (2019).(single column fitting image).

In order to obtain detailed information of the observations, every participant was asked to describe where the sighting took place. In Austria, 15 (52%) observations were made at trap nests and another 8 (28%) during interactions with the following plant taxa (Fig 2): 2 x *Lavandula* sp., and once at *Wisteria* sp., *Lathyrus latifolius*, *Eupatorium fistulosum*, *Centaurea scabiosa*, *Betonica officinalis* and *Vitex agnus-castus*. Another 3 (10%) specimens were observed nesting in holes in wooden furniture and roof beams. One specimen (3%) got entangled in the hair of a participant. Finally, 2 (7%) specimens were found dead, one on the ground of a street and the other drowned in a pool (S2 Table and S1 Fig). Two participants recorded agonistic behavior in terms of nest evacuations of other wild bees, one *Osmia cornuta* and another undetermined specimen.

### Citizen science in Switzerland and Liechtenstein

**Fig 3:**
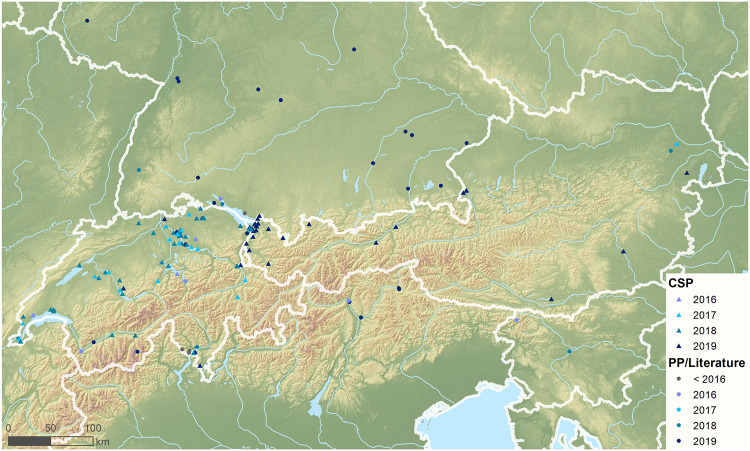
Occurrence data gathered by citizen scientists displayed together with reports of *Megachile sculpturalis* found in literature and nature platforms from Germany [42,55], Italy [52] and Slovenia [45]. (2-column fitting image).

Besides four observations published in previous literature [41–43,54] and eight reports documented on public platforms (S4 Table), the presented CSP added another 80 records since 2016 in Switzerland and one for Liechtenstein. The records stem from 67 participants, which reported occurrences from 62 cities and 17 federal states in Switzerland (Fig 3; S3 Table and S2 Fig). Six reports (9%) were received in 2018, whereas 61 observations were collected in the following year in 2019. In eight cities (Bern, Frauenfeld, Genf, Lausanne, Meilen, St. Margarethen, Sargans, Zurich), the species was found at least twice. Furthermore, seven years after its first observation in Switzerland, the species has not been detected at higher altitudes of the Swiss alpine massif. *Megachile sculpturalis* was observed exclusively within the big valleys of the Rhône in the south-east and the Rhine in the west with lower altitudes (660 m ASL; 637 m ASL). The observations were made at altitudes from 217 m ASL (Gerra) to 1048 m ASL (Geneva) with an average elevation of 483.0 ± 115.7 m ASL. At the second alpine region (Jura) of eastern Switzerland, which also reaches into France, *M*. *sculpturalis* was not detected on both sites of the border [39].

The main source connecting non-professionals with the presented CSP was the company “Wildbiene+Partner” with 69% of all reports, followed by 14% of the observations based on the published articles at the Entomological Club Bern, 4% on the Facebook page of the Entomological Club Zurich and another 4% as a result of an article in a local newspaper “Meilener Zeitung” (15.8.2019). For the remaining 9% of the participants, the source raising attention for the described citizen science project was unknown.

Due to this distribution, most of the Swiss participants owned a bee hotel and in total 63 (94%) observations were made at artificial nesting sites such as bee hotels and trap nests, of which 45 observations were contributed by customers of "Wildbiene+Partner". Only 2 (3%) observations were made on plants: *Lavandula* sp. and *Lathyrus latifolius*. One female was observed nesting in an abandoned cavity built by females of the genus *Xylocopa* sp. and one specimen was found at the ground alive. Three observations where the species evacuated nests of *Osmia cornuta* were recorded (S1 and S2 Videos).

### Temporal development of spatial extent and estimated distribution range

**Fig 4:**
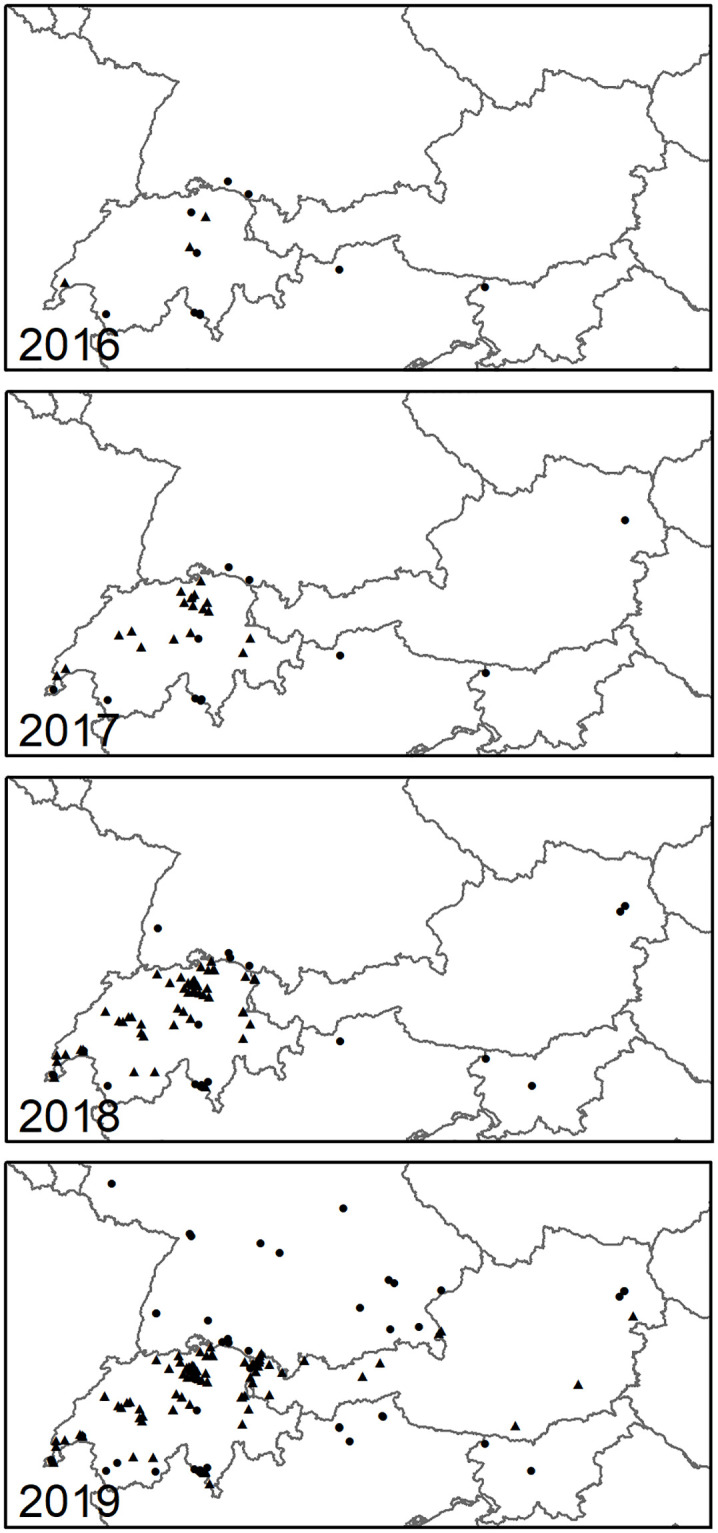
Localities split according to years from 2016 to 2019. New data are shown as triangles and data from literature as dots. We note an increase of localities from year to year in all regions. (single column fitting image).

**Fig 5:**
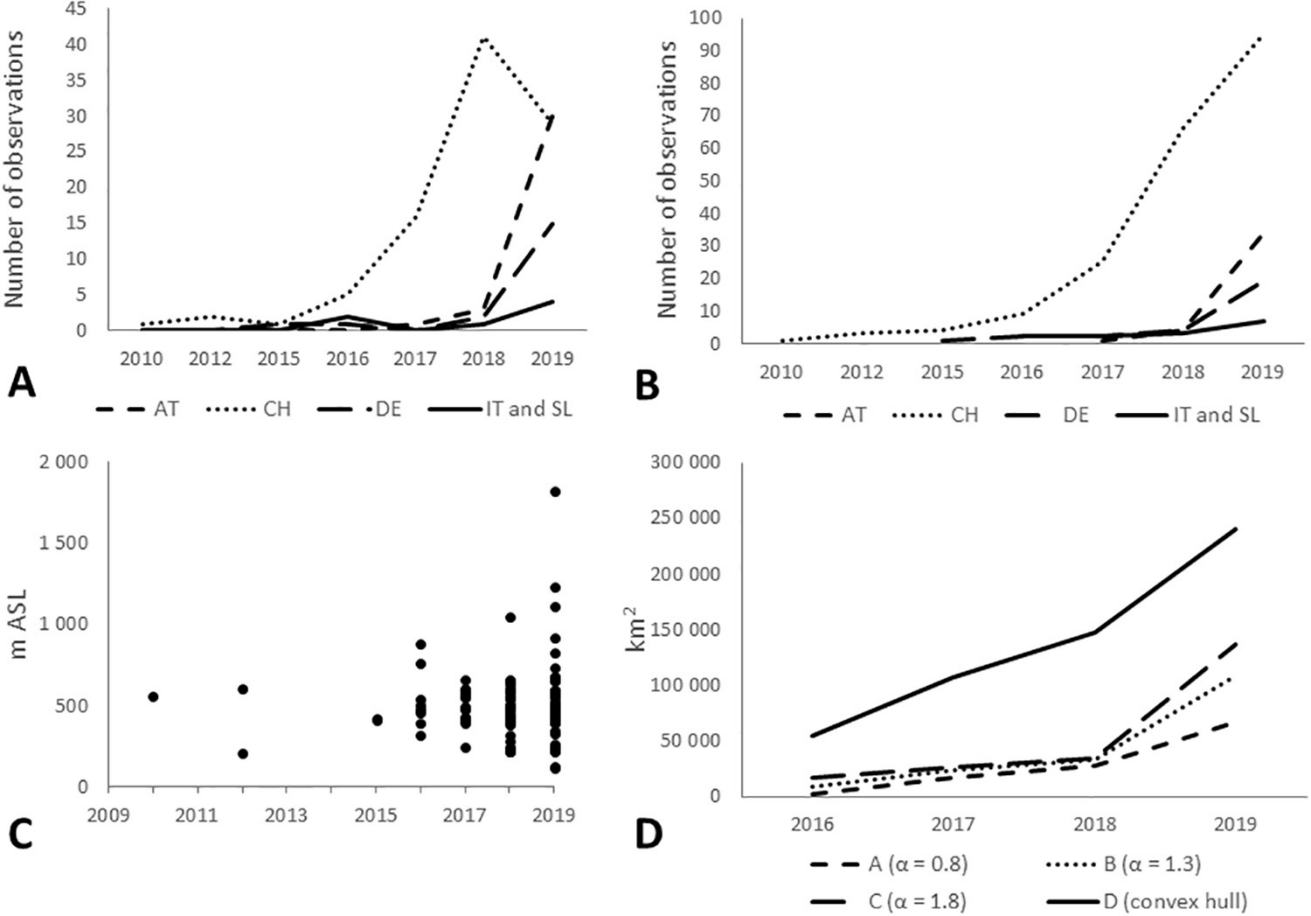
A: Localities per year and country gathered by the present citizen science project; B: All data points between 2010 and the respective year; C: Respective altitude of the localities per year indicates a potential increase of the ecological range; D: Estimated distribution area of *Megachile sculpturalis* compared concerning approach and year. (2-column fitting image).

**Fig 6:**
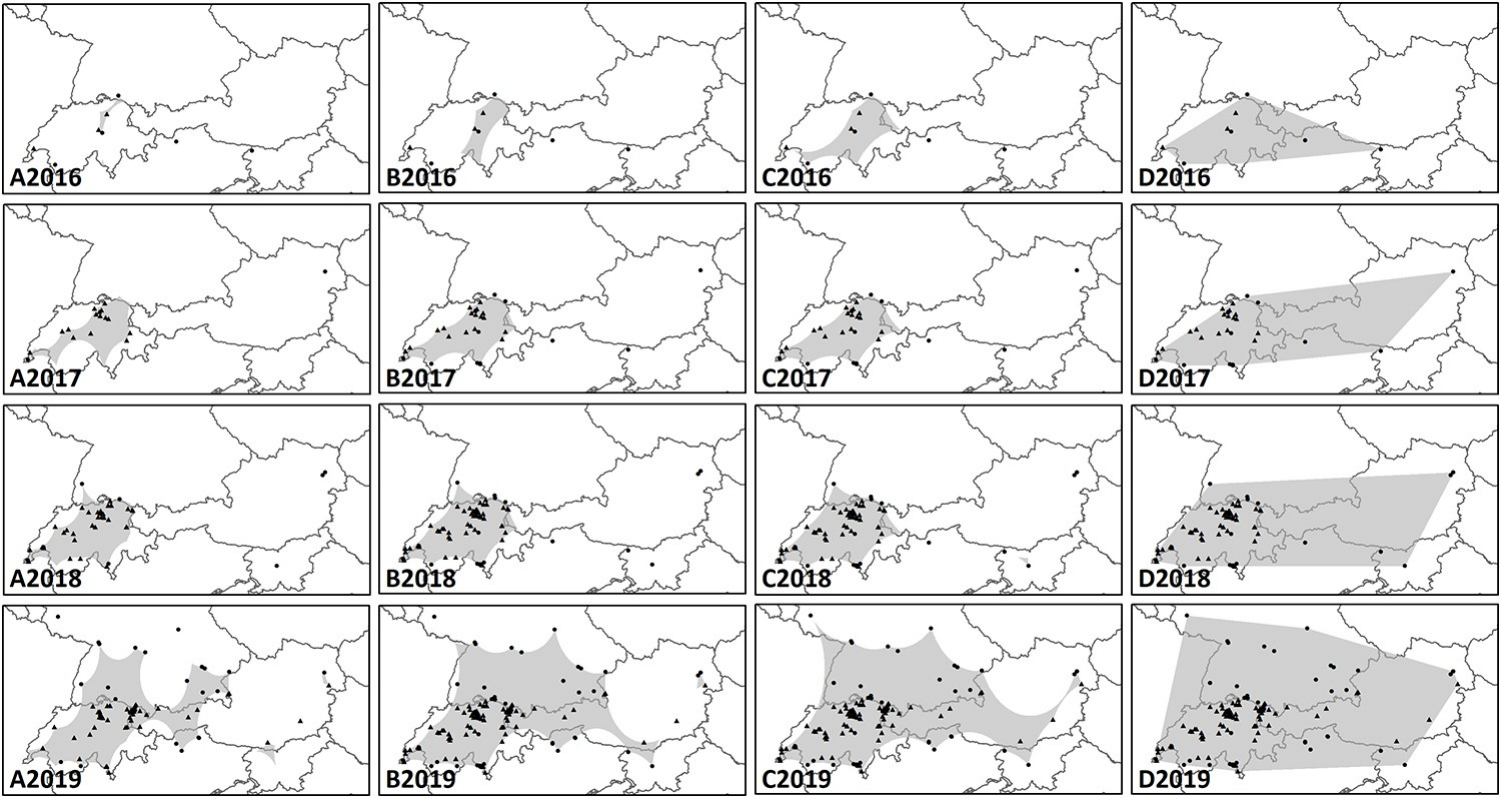
Estimated distribution area of *Megachile sculpturalis* in Switzerland, Liechtenstein and Austria based on alpha-convex hulls with A: α = 0.8; B: α = 1.3; C: α = 1.8 and D: minimum convex polygons. (2- column fitting image).

From 2010 to 2016 only few records existed for the alpine region. According to the present data set, reports of the species increased with each year within the examined region (Fig 4). While newly recorded reports from Switzerland are dating back to 2016, the majority of the Austrian observations are recorded in 2019. Furthermore, occurrence data implicate that the species expanded its range throughout the period under review within the alpine area (Fig 5). In Switzerland, occurrence data increased until 2018 and dropped in 2019, while in Austria, Germany, Italy and Slovenia, records increased rapidly. This is indicated using number of observations geographically and as altitude. All our results indicated *M*. *sculpturalis* an increase reconstructed distribution range with the different approaches used (Table 2.; Figs 5D and 6). Using the conservative approach (approach A, α-convex hull, α = 0.8) the reconstructed range size increased 48-fold between ≤2016 and ≤2019, with the largest increase from ≤2016 to ≤2017. The estimated distribution for ≤2019 indicated three hulls. The largest hull stretched from western Switzerland to Salzburg and from southern Germany to southern Switzerland and northern Italy but featured a narrow connex around Tyrol. The two smaller hulls were located in eastern Austria and in northern Slovenia/southern Austria.

The intermediate approach (approach B, α-convex hull, α = 1.3) displayed an approximately 13-fold of total area from ≤ 2016 to ≤ 2019 with the highest increase from ≤ 2018 to ≤ 2019. The estimated distribution predicted by approach B was formed from two unconnected hulls. The larger hull spanned from western Switzerland to eastern Slovenia and from southern Germany to southern Switzerland, northern Italy and northern Slovenia. It included a considerable area without evidenced observations in southern Germany and a narrow connex between Salzburg, northern Italy, southern Austria and northwestern Slovenia. The smaller hull indicated a disconnected hull in eastern Austria.

Under the radical approach (approach C, α-convex hull, α = 1.8), the estimated distribution approximately 8-folded area from ≤2016 to ≤2019 with the highest increase from ≤2018 to ≤2019. It led to one shared hull instead of multiple disconnected hulls, stretching from western Switzerland to eastern Austria and from southern Germany to southern Switzerland, northern Italy and northwestern Slovenia, and encompassing two areas without evidenced observations in southern Germany and central Austria as well as a narrow connex from southern to eastern Austria.

The generalized approach (approach D, minimum convex polygon) by nature led to a shared convex hull between all data points, independent from any encompassed areas without evidenced observations. The area approximately increased by the 4-fold from ≤2016 to ≤2019, with the largest increase from ≤2016 to ≤2017 (Table 2).

**Table 2:** Area of estimated species distribution [km^2^] were reconstructed with minimum convex polygons and alpha-convex hulls. Alpha hulls for each year were calculated using three different α values. If not indicated otherwise, estimated ranges were formed from a single hull. Areas consisting of two * and three ** hulls are marked.

year	2016	2017	2018	2019
hull type				
α = 0.8 [km^2^]	1393*	17367	27600	67116**
α = 1.3 [km^2^]	8516	23859	32328	108391*
α = 1.8 [km^2^]	17265	26161	34797*	136120
convex hull [km^2^]	54533	106359	147302	240391

## Discussion

There are around 80 recorded bee species introduced to a new habitat around the world, whereby the genus *Megachile* is represented most frequently. The majority of all non-native bees were taken accidently to their new habitat, while only a few (18%) species were introduced deliberately as providers of honey or pollination services for crop [34]. A lot of research concentrated on these bees species, such as *Apis mellifera* Linnaeus, 1758 or *Bombus terrestris* (Linnaeus, 1758) outside their historical range, but there is a major lack of knowledge concerning other non-managed and especially invasive bees [83–86].

Here, we present 111 new reports of *M*. *sculpturalis* from three alpine countries, Switzerland, Liechtenstein and Austria between 2016 and 2019 surveyed by a citizen science project. In Austria, only two published locations were registered in 2017 and 2018 for the exotic bee species. We added another 29 locations in 20 areas from 30 observations. A similar picture is presented for Switzerland, where four findings were published in previous literature [41–43,54] and another eight observations have been stored on online platforms (iNaturalist, info fauna CSCF). With the presented CS monitoring program, we gathered another 81 observations from 67 locations for Switzerland and Liechtenstein. The report from Liechtenstein was the first recorded observation from this country.

### Distribution and range expansion of *M*. *sculpturalis*

Long-distance introductions of species are mainly caused by direct or indirect human activities, which often relate to an economic purpose. From there, the newly introduced organism disperses diffusively colonizing the new range. Diffusion dispersal describes stepwise small-scale dispersal patterns, whereas long distances had been overcome most likely by jump-dispersal, which can be carried out passively mediated by a transportation vector [87–89]. The present situation of *M*. *sculpturalis* in Europe could be explained by introduction via ships to a harbor close to the location of the first record in Aullach, France, probably Marseille. From there, the species expanded its range within Europe [39,40].

After its first discovery in northern Italy close to the Swiss border, *M*. *sculpturalis* was also found north at the Swiss part of the Lake Maggiore area [41]. Three years after its first observation south of the Alps, *M*. *sculpturalis* occurred north, first in Altendorf in the canton Schwyz [43] and in the city of Zurich [42]. As shown here, a few years later the species was recorded in many other regions in Switzerland, except the mountain massifs of the Alps and Jura.

In Austria the situation differs from that in Switzerland as the received records of the species reflect an early invasion stage and a disjunct distribution. At the Lake Constance area, a population of the species was already discovered in 2015 [55]. Therefore, *M*. *sculpturalis* was expected to be found in western Austria subsequently. Except the records along the Swiss and German border, observations in western Austria were isolated, separated from populations in Bavaria and North-Italy, for example by the Arlberg pass, Bavarian alps and Brenner pass [50,52]. These topographical barriers between Germany, Austria and Italy are connected by heavily used traffic and transportation routes [90]. Because intermediate records are missing, and the Alps constitute a natural barrier, this distribution pattern indicate that the species might have been transported between the localities.

*Megachile sculpturalis* was also reported in 2016 from the Trenta valley in northern Slovenia [45] and 2018 in Ljubljana [91]. Because of these findings, we expected to receive records of *M*. *sculpturalis* from southern regions of Austria, which was verified by citizen scientists reporting the species in Klagenfurt and Graz in 2019. Those occurrences are consistent with dispersal by active flight as the localities are not separated by a topographical barrier and within a distance of 48 km to Klagenfurt.

According to the distribution and timing of records, the dispersal within European alpine countries happened remarkably fast with an increase in area estimated as minimal 4-fold and maximal 48-fold from ≤ 2016 to ≤ 2019. While the trend remained the same for all reconstructions, total area and shape of the estimated distributions from all data varied remarkably between the four estimations. Minimum convex polygons are designed to incorporate all observations and therefore include also large gaps without evidenced observations. Minimum convex polygons consider all locations, but tend to overestimate the actual area when it consists of disjunct parts [62]. This can reflect also dispersal via large distances. In accordance to Burgman and Fox [62], the reconstructions calculated using α-convex hulls can be interpreted as more reliably. They allow for disconnected shapes, thus constitute a flexible method for estimating distribution ranges solely from presence data [59]. However, disruptive ranges that occur during range expansion composing of stepwise short-distance dispersal mechanisms and long-distance jump dispersal incidents are also not well explained. The most distinctive outlier in this respect are the observations around Vienna in 2017. This is 280 km distant from the location in Slovenia (Trenta) and 430 from South-Tyrol (Meran) in the respective year. It is 280 km away from the closest report in Hungary from 2015 [46]. The closest locality in Austria from 2018 is 550 km distant to Vienna. The occurrence in Vienna can either be explained by a long-distance dispersal event from the invasive range west from the locality, or more likely from the closer locality in Slovenia or Hungary. The record had therefore been treated as an outlier and could not be included in our study. Taking these records into consideration, Austria is seemingly colonized from different directions and via short and long-distance dispersal mechanisms.

Similarly, to southern Austrian, also western Austria is potentially connected via short-distance dispersal with the previously established populations in Switzerland. The locality in Vienna is best explained by long distance dispersal. However, the observation indicates the possibility that two different invasion fronts are currently colonizing Europe and are coming into contact within our study region. This is a common feature of biological invasion [92]. Future work will investigate this possibility further. Regardless, of the occurrence explained by jump dispersal from within the main invasive range or as multiple introduction of the species into Europe, the velocity with which the high distances are covered during the colonization show the high invasive potential of the species for Europe. The exceptional fast dispersal overcoming topographical barriers between the localities of *M*. *sculpturalis* in Austria support the hypothesis that its dispersal within Europe follows human movement and transportation routes. Additionally, the recent finding of *M*. *sculpturalis* at the Crimea (Russia) in 2019, indicates a further human assisted introduction as the nearest report is 1130 km away in Hungary, a distance too long for a natural spread within three years [47]. This is in line with previous studies demonstrating that the accelerating rates of international transportation are responsible for the unintentional introduction of a variety of taxonomic groups [29,93–95].

### Potential biases and further potential of CS data

Citizen science programs are often described as a popular and practical way investigating species range shifts, migration patterns and population trends on a reasonable geographical and temporal scale [3]. Nevertheless, many CSP approaches were criticized due to a weak accuracy of species identification based on photos rather than on specimens for some groups [11,96]. This bias might be an issue for some programs and the differentiation of most wild bees is certainly a case for experts [97,98]. Yet, the target species with its distinctive appearance led to a high prevalence of accurate identified observations of the volunteers. Additionally, the observations were validated by designated experts. This procedure enabled the volunteers to contribute to high-quality species distribution data sets of *M*. *sculpturalis*.

Another point of criticism of CS programs that are focusing on species distribution data are spatial biases due to uneven sampling [99]. In contrast, recent projects show that CS programs are often less spatially biased than long-term studies or global platforms utilized mainly by experts [4,98]. Furthermore, it has been shown that CSP are fast and effective tools investigating distributional ranges of elusive species. A study demonstrated that within only two years of data gathering by citizen scientists, range size estimates (extend of occurrence and area of occupancy) were comparable to a long-term study [71]. However, during this study there might be a high prevalence of reports originated from human settlements resulting in an urban bias [4] as the present data set lacks occurrences in remote areas. Such uninhabited areas are often under-sampled in the frame of CSPs, which can lead to misinterpretation of the distribution data concerning population size, abundancy and habitat preferences [2]. This means, that even though the species did not occur in certain areas, it might just has not been detected yet.

Nevertheless, every species thrives within a limited range of environmental conditions, where activity and reproduction ensure its survival [100]. Wild bees require an appropriate habitat, a nesting site and pollen resource suppling their offspring [101]. In respect of appropriate nesting sites, the cavity nesting species was mainly observed at artificial nests, which are set up at human settlements of different population densities in their gardens or balconies. Besides, previous literature described the requirements of exotic plants as feeding resource for a successful colonization and establishment of a new area [40,48]. As a polylectic species, *M*. *sculpturalis* feeds on many different plant species with a preference for pollen of *Sophora japonica*, which occurs in Europe mainly in urban environments as ornamental trees in parks and gardens [31,40,50,102]. Therefore, the prevalence of the urban occurrence of *M*. *sculpturalis* might be based on a spatial bias, but likewise it might reflect the synanthropy of the species. Anyhow, further knowledge of the ecological requirements, especially its pollen resource, is required in order to clarify the underlying relationship.

Besides spatial biases, also temporal biases of ecological CSP resulting in data quality problems have to be addressed [103,104]. By calling citizens to watch out and report a target species, a linkage between advertising the project and a population progression can be problematic interpreting the data. Yet, participants were asked to clarify in which year the bee was recognized for the first time. Although the CSP was advertised most intensively in the year 2019 and most reports were gathered in the respective year, occurrences date back to 2016. Bias of the data set might occur by difficulties to notice unexpected objects in their environment due to attention-demanding tasks [“inattentional blindness”, 105–107]. Animated stimuli, like flying bees, are attracting attention and are therefore frequently identified [108]. Because most reports were made at artificial nesting sites, which were consciously observed by the citizen scientists, this effect should not play a major role.

But besides these potential difficulties of CS approaches, the popularity in ecology studies increased, although the search for invasive species by citizen scientists on a broad geographical scale is not a new invention [99]. By implementing computer-based programs and smartphones in CSP, the diversity and popularity even increased [98,109,110]. Furthermore, using social media platforms and nature platforms for CSP has been helpful for getting in direct contact with participants answering questions and explaining instructions directly [49]. Citizen scientists get involved into research procedures and with their new insights, the public support of scientific studies develops [111]. Furthermore, with the increasing homogenization of the global biodiversity, it is more important than ever to raise awareness of the existence and consequences of non-native species [112,113]. Studies have shown that including a community with a certain interest into CSP, the outcome for the participants and the scientific investigations were particularly successful [3,12,114]. Additionally, these programs have the potential to inform the participants about conservation actions and the complexity of ecological networks [111,115]. As the majority of all observations of *M*. *sculpturalis* were made at artificial nesting sites, the volunteers showed a certain interest for wild bees beforehand by hosting small ecosystems in their backyard or balcony.

It was our aim to sustainably integrate our participants into the project, thus, the participants got feedback on their observations directly after the first contact, as outlined by the guidelines of CSP by Silvertown [99]. Moreover, the citizen scientists received a monthly newsletter for the duration of the project with informative facts about wild bees, conservation actions and regular updates of the project. With this procedure, the participants did not just deliver data, but the project added an educational value for the local biodiversity.

### Consequences of the introduction of *M*. *sculpturalis* on the native bee fauna

Introduced bees are able take over an important role within pollination networks in their new habitat [34,112], but they might as well have negative impacts on native fauna and flora. Introduced wild bees can act as competitors for native species in terms of floral resources and nest sites [116,117]. By investigating artificial nests as study sites, a negative correlation between the occurrence of *M*. *sculpturalis* and native bees was recently detected [118].

Although Geslin et al. [118] mentioned that this worryingly trend was probably caused by its territorial behavior against the local bee fauna not a direct competition of nest sites, several incidents of nest evacuations were recorded. In the US, *M*. *sculpturalis* was observed destroying nests of the native wild bee *Xylocopa virginica* Linnaeus, 1771 [119,120]. In Europe *M*. *sculpturalis* was observed clearing out grasshoppers of the species *Meconema meridionale* Costa, 1860 from a wasp’s nest *Isodontia mexicana* (Saussure, 1867) in 2017, which is also an invasive insect arriving at central Europe five decades ago [121]. The wasp stored the grasshopper as prey for its larvae before *M*. *sculpturalis* evacuated the whole nest material for its own use [50]. In our study in total four participants of the CSP observed the species clearing out several nests of mason bees cleaning and rebuilding its nests in 2018 and 2019 (videos showing a *M*. *sculpturalis* individual removing multiple pupae of *Osmia sp*. from breeding cavities are provided as S1 and S2 Videos). Furthermore, *M*. *sculpturalis* was observed using material from the entrance plug of the cavity of a native wild bee`s nest leaving the nest open. To determine if the above described behaviors are single events or validate the hypothesis of direct or indirect competition between *M*. *sculpturalis* and native cavity nesters requires evaluation of the incidence and circumstances of apparent nest evacuations, which might be achieved with the help of further observations from citizen scientists in future. The presented results highlight the urgency of an international consortium focusing on introduced and potentially invasive wild bees to discuss and assess further action and management plans.

## Conclusion

Citizens participated in a fundamental biodiversity research project with the aim to gather ecological data by observing, measuring and recording the occurrence of an introduced wild bee. *Megachile sculpturalis* has been reported in most areas in Switzerland and was also found in Liechtenstein and Austria. The dispersal is remarkably fast, indicating different dispersal mechanisms: diffusion-dispersal and jump-dispersal modes. Due to long-distance introductions within this short period of time, we assume that the dispersal partly stems from human assisted transportation of *M*. *sculpturalis* on the major traffic routes across Europe.

## Supporting information

S1 Table List of supporting institutions, who published calls for reports collaborating at a citizen science project focusing on *Megachile sculpturalis*. (PDF)

S2 Table Detailed information on the observational data gathered in Austria by citizen scientists. (PDF)

S3 Table Detailed information on the observational data gathered in Switzerland and Liechtenstein by citizen scientists. Abbreviation: nd = no data of adult individuals available as only the nest was observed.
(PDF)

S4 Table Occurrence data used for this study derived from literature or nature platforms. (PDF)

S1 Video *Megachile sculpturalis* clears out the nest of a mason bee. (MOV)

S2 Video *Megachile sculpturalis* clears out the nest of a mason bee. (MP4)

S1 Fig Detailed information on the observational data gathered in Austria by citizen scientists. (PNG)

S2 Fig Detailed information on the observational data gathered in Switzerland and Liechtenstein by citizen scientists. Abbreviation: nd = no data of adult individuals available as only the nest was observed.
(PNG)

## References

[1] Miller-Rushing A, Primack R, Bonney R. The history of public participation in ecological research. Frontiers in Ecology and the Environment. 2012;10(6):285–90.

[2] Dickinson JL, Zuckerberg B, Bonter DN. Citizen science as an ecological research tool: challenges and benefits. Annual Review of Ecology, Evolution, and Systematics. 2010;41(1):149–72.

[3] Chandler M, See L, Copas K, Bonde AMZ, López BC, Danielsen F, u. a. Contribution of citizen science towards international biodiversity monitoring. Biological Conservation. 2017;213:280–94.

[4] Sumner S, Bevan P, Hart A, Isaac NJB. Mapping species distribution in 2 weeks using citizen science. Insect Conservation and Diversity. 2019;12:382–8.

[5] Silvertown J, Cook L, Cameron R, Dodd M, McConway K, Worthington J, u. a. Citizen science reveals unexpected continental-scale evolutionary change in a model organism. PLoS ONE. 2011;6(4):e18927 10.1371/journal.pone.0018927 21556137 PMC3083392

[6] Tulloch AIT, Possingham HP, Joseph LN, Szabo J, Martin TG. Realising the full potential of citizen science monitoring programs. Biological Conservation. 2013;165:128–38.

[7] Pocock MJO, Tweddle JC, Savage J, Robinson LD, Roy HE. The diversity and evolution of ecological and environmental citizen science. PLOS ONE. 2017;12(4):e0172579 10.1371/journal.pone.0172579 28369087 PMC5378328

[8] Basset Y, Lamarre GPA. Toward a world that values insects. Science. 2019;364(6447):1230–1. 10.1126/science.aaw7071 31249044

[9] Lamarre GPA, Juin Y, Lapied E, Le Gall P, Nakamura A. Using field-based entomological research to promote awareness about forest ecosystem conservation. Nature Conservation. 2018;29:39–56.

[10] Deguines N, Julliard R, de Flores M, Fontaine C. The whereabouts of flower visitors: contrasting land-use preferences revealed by a country-wide survey based on citizen science. PLoS ONE. 2012;7(9):e45822 10.1371/journal.pone.0045822 23029262 PMC3446938

[11] Lye GC, Osborne JL, Park KJ, Goulson D. Using citizen science to monitor Bombus populations in the UK: nesting ecology and relative abundance in the urban environment. Journal of Insect Conservation. 2012;16(5):697–707.

[12] Graham JR, Tan Q, Jones LC, Ellis JD. Native Buzz: Citizen scientists creating nesting habitat for solitary bees and wasps. Florida Scientist. 2014;77(4):204–18.

[13] Prudic K, McFarland K, Oliver J, Hutchinson R, Long E, Kerr J, u. a. eButterfly: Leveraging massive online citizen science for butterfly conservation. Insects. 2017;8(2):1–12. 10.3390/insects8020053 PMC5492067 28524117

[14] Lander T. Network modelling, citizen science and targeted interventions to predict, monitor and reverse bee decline. PLANTS, PEOPLE, PLANET. 2020;2(2):111–20.

[15] Serret H, Deguines N, Jang Y, Lois G, Julliard R. Data quality and participant engagement in citizen science: comparing two approaches for monitoring pollinators in France and South Korea. Citizen Science: Theory and Practice. 2019;4(1):22.

[16] Brown MJF, Paxton RJ. The conservation of bees: a global perspective. Apidologie. 2009;40(3):410–6.

[17] Devictor V, Whittaker RJ, Beltrame C. Beyond scarcity: citizen science programmes as useful tools for conservation biogeography: Citizen science and conservation biogeography. Diversity and Distributions. 2010;16(3):354–62.

[18] Cardoso P, Erwin TL, Borges PAV, New TR. The seven impediments in invertebrate conservation and how to overcome them. Biological Conservation. 2011;144(11):2647–55.

[19] Le Féon V, Henry M, Guilbaud L, Coiffait-Gombault C, Dufrêne E, Kolodziejczyk E, u. a. An expert-assisted citizen science program involving agricultural high schools provides national patterns on bee species assemblages. Journal of Insect Conservation. 2016;20(5):905–18.

[20] Franklin J. Mapping species distributions: spatial inference and predictions. Cambridge University Press 2010;

[21] Potts SG, Biesmeijer JC, Kremen C, Neumann P, Schweiger O, Kunin WE. Global pollinator declines: trends, impacts and drivers. Trends in Ecology & Evolution. 2010;25(6):345–53. 20188434 10.1016/j.tree.2010.01.007

[22] Bellard C, Cassey P, Blackburn TM. Alien species as a driver of recent extinctions. Biology Letters. 2016;12(2):1–4. 10.1098/rsbl.2015.0623 PMC4780541 26888913

[23] Sánchez-Bayo F, Wyckhuys KAG. Worldwide decline of the entomofauna: A review of its drivers. Biological Conservation. 2019;232:8–27.

[24] Cooper C, Hochachka W, Dondt A. Contrasting natural experiments confirm competition between House Finches and House Sparrows. Ecology. 2007;88:864–8670. 10.1890/06-0855 17536703

[25] Eurad C, Boutin J-M, Roux D, Faivre B. Spatial dynamics of an invasive bird species assessed using robust design occupancy analysis: the case of the Eurasian collared dove (Streptopelia decaocto) in France. Journal of Biogeography. 2007;34:1077–86.

[26] Droege S. Introduced and Alien Bee Species of North America (North of Mexico). USGS science for a changing world. 2018.

[27] Roques A, Rabitsch W, Rasplus J-Y, Lopez-Vaamonde C, Nentwig W, Kenis M. Alien terrestrial invertebrates of Europe In: Handbook of Alien Species in Europe. Dordrecht: Springer Netherlands; 2009 S. 63–79.

[28] Heung-Sik L, Dong-Pyo R. Insect Fauna of Korea. Arthropoda: Insecta: Hymenoptera: Megachilidae Leafcutter Bees. 4. Aufl. Bd. 13. Korea: National Institute of Biological Resources, Ministry of Environment; 2013.

[29] Seebens H, Essl F, Dawson W, Fuentes N, Moser D, Pergl J, u. a. Global trade will accelerate plant invasions in emerging economies under climate change. Global Change Biology. 2015;21(11):4128–40. 10.1111/gcb.13021 26152518

[30] Magnum WA, Brooks RW. First Records of Megachile (Callomegachile) sculpturalis Smith (Hymenoptera: Megachilidae) in the Continental United States. Journal of the Kansas Entomological Society. 1997;70(2):140–2.

[31] Magnum WA, Sumner S. A survey of the North American range of Megachile (Callomegachile) sculpturalis, an adventive species in North America. Journal of the Kansas Entomological Society. 2003;76(4):658–62.

[32] Paiero SM, Buck M. The Giant Resin Bee, Megachile sculpturalis Smith, and other newly introduced and newly recorded native Megachilidae and Andrenidae (Apoidea) from Ontario. Journal of the Entomological Society Ontario. 2003;134:5.

[33] Parys K, Tripodi A, Sampson B. The Giant Resin Bee, Megachile sculpturalis Smith: New distributional records for the Mid- and Gulf-south USA. Biodiversity Data Journal. 2015;3:e6733. 10.3897/BDJ.3.e6733 PMC4678803 26696766

[34] Russo L. Positive and negative impacts of non-native bee species around the world. Insects. 2016;7(4):69. 10.3390/insects7040069 PMC5198217 27916802

[35] Sheffield CS, Dumesh S, Cheryomina M. Hylaeus puncatus (Hymenoptera: Colletidae), a bee species new to Canada, with notes on other non-native species. Journal of the Entomological Society Ontario. 2011;142:29–43.

[36] Stout JC, Kells AR, Goulson D. Pollination of the invasive exotic shrub Lupinus arboreus (Fabaceae) by introduced bees in Tasmania. Biological Conservation. 2002;106:425–34.

[37] Tonietto RK, Ascher JS. Occurrence of the old world bee species Hylaeus hyalinatus, Anthidum manicatum, A. oblongatum, and Megachile sculpturalis, and the native species Coelioxys banksi, Lasioglossum michiganense, and L. zophops in Illinois (Hymenoptera: Apoidea: Colletidae, Halictidae, Megachilidae). The Great Lakes Entomologist. 2008;41:200–3.

[38] Vereecken PNJ. Premières données sur la présence de l’abeille asiatique Megachile (Callomegachile) sculpturalis Smith (Hymenoptera, Megachilidae) en Europe. Osmia. 2009;3:4–6.

[39] Le Féon V, Aubert M, Genoud D, Andrieu-Ponel V, Westrich P, Geslin B. Range expansion of the Asian native giant resin bee Megachile sculpturalis(Hymenoptera, Apoidea, Megachilidae) in France. Ecology and Evolution. 2018;00:1–9. 10.1002/ece3.3758 PMC5792562 29435230

[40] Quaranta M, Sommaruga A, Balzarini P, Felicioli A. A new species for the bee fauna of Italy: Megachile sculpturalis continues its colonization of Europe. Bulletin of Insectology. 2014;67(2):287–293.

[41] Amiet F. Die Blattschneiderbiene Megachile sculpturalis Smith, 1853 (Hymenoptera, Apidae) nun auch in der Schweiz. Entomo Helvetica. 2012;5:157–9.

[42] Westrich P, Knapp A, Berney I. Megachile sculpturalis Smith 1853 (Hymenoptera, Apidae), a new species for the bee fauna of Germany, now north of the Alps. Eucera. 2015;9:3–10.

[43] Dillier F-X. Eingeschleppte Asiatische Mörtelbiene Megachile sculpturalis Smith, 1853 (Hymenoptera, Apidae) erstmals nördlich der Alpen gesichtet. Entomo Helvetica. 2016;(9):153–6.

[44] Wiesbauer H. Wilde Bienen. Biologie- Lebensraumdynamik am Beispiel Österreich-Artenporträts. 1. Aufl. Bd. 1. Stuttgart: Ulmer Verlag; 2017. 376 S.

[45] Gogala A, Zadravec B. First record of Megachile sculpturalis Smith in Slovenia (Hymenoptera: Megachilidae). Acta entomologica Slovenica. 2018;26:79–82.

[46] Kovács T. Megachile sculpturalis Smith, 1853 in Hungary (Hymenoptera, Megachilidae). Folio Historico-Naturalia Musei Matraensis. 2015;39:73–6.

[47] Ivanov SP, Fateryga AV. First record of the invasive giant resin bee Megachile (Callomegachile) sculpturalis Smith, 1853 (Hymenoptera: Megachilidae) in the Crimea. Far Eastern Entomologist. 2019;395:7–13.

[48] Aguado O, Hernández-Castellano C, Bassols E, Miralles M, Navarro D, Stefanescu C, u. a. Megachile (Callomegachile) sculpturalis Smith, 1853 (Apoidea: Megachilidae): a new exotic species in the Iberian Peninsula, and some notes about its biology. Butlletí de laInstitució Catalana d’Història Natural. 2018;82:157–62.

[49] Maher S, Manco F, Ings TC. Using citizen science to examine the nesting ecology of ground-nesting bees. Ecosphere. 2019;10(10).

[50] Westrich P. Faszination Wildbienen, Forschungsprojekte: Megachile sculpuralis. wildbienen.info. 2018.

[51] Hinojosa-Diaz I, Yanez-Ordonez O, Chen G, Perterson T, Engel M. The North American invasion of the Giant Resin Bee (Hymenoptera: Megachilidae). Journal of Hymenoptera Research. 2005;14(1):69–77.

[52] Guariento E, Lanner J, Staggl MA, Kranebitter P. Megachile sculpturalis (Smith, 1853) (Hymenoptera: Megachilidae), the giant resin bee new for South Tyrol with a newly described plant species interaction. Gredleriana. 2019;19.

[53] Pachinger B. Die Asiatische Mörtelbiene in Österreich. Natur&Land. 2018;2/2018:56.

[54] Rickenbach F, Sprecher E. Neue Bestäuberin von Bienenbäumen: die Asiatische Mörtelbiene. Schweizerische Bienen-Zeitung. 2018;9:15–7.

[55] Westrich P. Neues zur Ausbreitung der Mörtelbiene Megachile sculpturalis Smith 1853 (Hymenoptera: Anthophila) in Deutschland—Stand Oktober 2019. Eucera. 2020;14:12–5.

[56] Stadt Wien—ViennaGIS. Geodatenviewer der Stadtvermessung Wien. Magistrat der Stadt Wien (MA41), 2015. Vienna; 2016. (ESRI).

[57] R Core Developmemnt Team. RStudio: Integrated Development for R. [Internet]. Boston: RStudio, Inc.; 2018. http://www.rstudio.com/.

[58] Copernicus—The European Earth Observation Programme. Europe`s eyes on Earth (EEA). 2013.

[59] Capinha C, Pateiro-López B. Predicting species distributions in new areas or time periods with alpha-shapes. Ecological Informatics. 2014;24:231–7.

[60] Pateiro-López B, Rodríguez-Casal A. Generalizing the Convex Hull of a Sample: The R Package alphahull. Journal of Statistical Software [Internet]. 2010;34(5). http://www.jstatsoft.org/v34/i05/

[61] Bland LM, Keith DA, Miller RM, Murray NJ, Rodríguez JP, Herausgeber. Guidelines for the application of IUCN Red List of ecosystems categories and criteria [Internet]. Gland, Switzerland: IUCN International Union for Conservation of Nature; 2015. https://portals.iucn.org/library/sites/library/files/documents/2016-010.pdf

[62] Burgman MA, Fox JC. Bias in species range estimates from minimum convex polygons: implications for conservation and options for improved planning. Animal Conservation. 2003;6(1):19–28.

[63] Edelsbrunner H, Kirkpatrick D, Seidel R. On the shape of a set of points in the plane. IEEE Transactions on Information Theory. 1983;29(4):551–9.

[64] Rosser N, Phillimore AB, Huertas B, Willmott KR, Mallet J. Testing historical explanations for gradients in species richness in heliconiine butterflies of tropical America: Diversification of butterflies. Biological Journal of the Linnean Society. 2012;105(3):479–97.

[65] Maes D, Isaac N, Harrower C, Collen B, van Strien A, Roy D. The use of opportunistic data for IUCN Red List assessments [in special issue: Fifty years of the Biological Records Centre]. Biological Journal of the Linnean Society. 2015;115(3):690–706.

[66] Worth JRP, Williamson GJ, Sakaguchi S, Nevill PG, Jordan GJ. Environmental niche modelling fails to predict Last Glacial Maximum refugia: niche shifts, microrefugia or incorrect palaeoclimate estimates? Global Ecology and Biogeography. 2014;23(11):1186–97.

[67] Rabosky DAR, Cox CL, Rabosky DL, Title PO, Holmes IA, Feldman A, u. a. Coral snakes predict the evolution of mimicry across New World snakes. Nat Commun. 2016;7(1):1–9. 10.1038/ncomms11484 PMC4858746 27146100

[68] Chacón-Madrigal E, Wanek W, Hietz P, Dullinger S. Is local trait variation related to total range size of tropical trees? Delzon S, Herausgeber. PLOS ONE. 2018;13(3):e0193268 10.1371/journal.pone.0193268 29513689 PMC5841763

[69] Cerasoli F, Thuiller W, Guéguen M, Renaud J, D’Alessandro P, Biondi M. The role of climate and biotic factors in shaping current distributions and potential future shifts of European Neocrepidodera (Coleoptera, Chrysomelidae). Insect Conservation and Diversity. 2020;13(1):47–62.

[70] Hui C, Richardson DM, Robertson MP, Wilson JRU, Yates CJ. Macroecology meets invasion ecology: linking the native distributions of Australian acacias to invasiveness. Diversity and Distributions. 2011;17(5):872–83.

[71] Zapponi L, Cini A, Bardiani M, Hardersen S, Maura M, Maurizi E, u. a. Citizen science data as an efficient tool for mapping protected saproxylic beetles. Biological Conservation. 2017;208:139–45.

[72] García-Roselló E, Guisande C, Manjarrés-Hernández A, González-Dacosta J, Heine J, Pelayo-Villamil P, u. a. Can we derive macroecological patterns from primary Global Biodiversity Information Facility data?: Macroecological patterns and GBIF data. Global Ecology and Biogeography. 2015;24(3):335–47.

[73] Pagel J, Treurnicht M, Bond WJ, Kraaij T, Nottebrock H, Schutte-Vlok A, u. a. Mismatches between demographic niches and geographic distributions are strongest in poorly dispersed and highly persistent plant species. Proceedings of the National Academy of Sciences. 2020;117(7):3663–9. 10.1073/pnas.1908684117 PMC7035498 32029599

[74] Matthews F. A review of the population and conservation status of British mammals. London: The Mammal Society; 2018. 699 S. (Natural England Joint Publication).

[75] Sundaram M, Donoghue MJ, Farjon A, Filer D, Mathews S, Jetz W, u. a. Accumulation over evolutionary time as a major cause of biodiversity hotspots in conifers. Proceedings of the Royal Society B: Biological Sciences. 2019;286(1912):20191887 10.1098/rspb.2019.1887 31594500 PMC6790781

[76] Pebesma E. Simple Features for R: Standardized Support for Spatial Vector Data. The R Journal. 2018;10(1):439–46.

[77] Pebesma E, Bivand RS. Classes and Methods for Spatial Data: the sp Package. 2005;5(2):1–21.

[78] European Digital Elevation Model (EU-DEM), version 1.1. 1.1. European Environment Agency (EEA) Copernicus programm; 2016.

[79] EU-Hydro River Network Database, Version 1.0. 1.0. European Environment Agency (EEA) Copernicus programm; 2019.

[80] Rivers + lake centerlines. 4.1. Natural Earth—Free vector and raster map data; 2019.

[81] eurostat. Eurographics—Countries 2016. eurostat; 2016. (eurostat).

[82] Federal office of Topography swisstopo. swissBoundaries3D. 3D Aufl. Wabern: Schweizerische Eidgenossenschaft; 2019.

[83] Dafni A, Kevan P, Gross CL, Goka K. Bombus terrestris, pollinator, invasive and pest: An assessment of problems associated with its widespread introductions for commercial purposes. Applied Entomology and Zoology. 2010;45(1):101–13.

[84] Goulson D. Impacts of non-native bumblebees in Western Europe and North America. Applied Entomology and Zoology. 2010;45(1):7–12.

[85] Morales CL, Arbetman MP, Cameron SA, Aizen MA. Rapid ecological replacement of a native bumble bee by invasive species. Frontiers in Ecology and the Environment. 2013;11(10):529–34.

[86] Paini DR. Impact of the introduced honey bee (Apis mellifera) (Hymenoptera: Apidae) on native bees: A review. Austral Ecology. 2004;29(4):399–407.

[87] Sakai AK, Allendorf FW, Holt JS, Lodge DM, Molofsky J, With KA, u. a. The population biology of invasive species. Annual Review of Ecology and Systematics. 2001;32(1):305–32.

[88] Bossenbroek JM, Kraft CE, Nekola JC. Prediction of long-distance dispersal using gravity models: Zebra mussel invasion of inland lakes. Ecological Applications. 2001;11(6):11.

[89] Trakhtenbrot A, Nathan R, Perry G, Richardson DM. The importance of long-distance dispersal in biodiversity conservation. Diversity and Distributions. 2005;11(2):173–81.

[90] Land Tirol. Verkehr in Tirol—Bericht 2017. Sachgebiet Verkehrsplanung [Internet]. 2017. https://www.tirol.gv.at/fileadmin/themen/verkehr/verkehrsplanung/downloads/verkehrsberichte/VB_2017_web.pdf

[91] Global Biodiversity Information Facility. GBIF Occurrence Download [Internet]. gbif. 2019. https://www.gbif.org/occurrence/download/0004715-191105090559680

[92] Roman J, Darling J. Paradox lost: genetic diversity and the success of aquatic invasions. Trends in Ecology & Evolution. 2007;22(9):454–64. 17673331 10.1016/j.tree.2007.07.002

[93] Pysek P, Jarosik V, Hulme PE, Kuhn I, Wild J, Arianoutsou M, u. a. Disentangling the role of environmental and human pressures on biological invasions across Europe. Proceedings of the National Academy of Sciences. 2010;107(27):12157–62. 10.1073/pnas.1002314107 PMC2901442 20534543

[94] Werner D, Kronefeld M, Schaffner F, Kampen H. Two invasive mosquito species, Aedes albopictus and Aedes japonicus japonicus, trapped in south-west Germany, July to August 2011. Rapid communications. 2012;17(4). 10.2807/ese.17.04.20067-en 22297138

[95] Neteler M, Metz M, Rocchini D, Rizzoli A, Flacio E, Engeler L, u. a. Is Switzerland Suitable for the Invasion of Aedes albopictus? PLoS ONE. 2013;8(12):e82090 10.1371/journal.pone.0082090 24349190 PMC3862574

[96] Lewandowski E, Specht H. Influence of volunteer and project characteristics on data quality of biological surveys. Conservation Biology. 2015;29(3):713–23. 10.1111/cobi.12481 25800171

[97] Schmidt S, Schmid-Egger C, Morinière J, Haszprunar G, Hebert PDN. DNA barcoding largely supports 250 years of classical taxonomy: identifications for Central European bees (Hymenoptera, Apoidea *partim*). Molecular Ecology Resources. 2015;15(4):985–1000. 10.1111/1755-0998.12363 25588628

[98] Suzuki-Ohno Y, Yokoyama J, Nakashizuka T, Kawata M. Utilization of photographs taken by citizens for estimating bumblebee distributions. Scientific Reports. 2017;7(1):e11215. 10.1038/s41598-017-10581-x PMC5594003 28894157

[99] Silvertown J. A new dawn for citizen science. Trends in Ecology & Evolution. 2009;24(9):467–71. 19586682 10.1016/j.tree.2009.03.017

[100] Chase JM, Liebold MA. Ecological niches: linking classical and contemporary approaches. University of Chicago Press; 2003 212 S.

[101] Mazzucco K, Mazzucco K, Mazzucco R, Dieckmann U. Wege der Mikroevolution und Artbildung bei Bienen (Apoidea, Hymenoptera): Populationsgenetische und empirische Aspekte. Densia. 2007;(20):617–85.

[102] Andrieu-Ponel V, Ponel P, Le Féon V, Geslin B, Duvallet G. À propos du comportement de butinage de Megachile sculpturalis Smith, 1853, en France méditerranéenne (Nîmes et Montpellier) (Hymenoptera, Megachilidae). Bulletin de la Société entomologique de France. 2018;123(1):49–54.

[103] Boakes EH, McGowan PJK, Fuller RA, Chang-qing D, Clark NE, O’Connor K, u. a. Distorted views of biodiversity: Spatial and temporal bias in species occurrence data. PLoS Biology. 2010;8(6):e1000385 10.1371/journal.pbio.1000385 20532234 PMC2879389

[104] Weigelhofer G, Pölz E-M. Data quality in citizen science projects: Challenges and solutions. Frontiers in Environmental Science. 2016;4 10.3389/fenvs.2016.00012 27642585 PMC5023020

[105] Simons DJ. Attentional capture and inattentional blindness. Trends in Cognitive Sciences. 2000;4(4):147–55. 10.1016/s1364-6613(00)01455-8 10740279

[106] Simons DJ, Chabris CF. Gorillas in our midst: sustained inattentional blindness for dynamic events. Perception. 1999;28:1059–74. 10.1068/p281059 10694957

[107] Mack A, Rock I, Inattentional Blindness. Cambridge, Massachusetts: MIT Press; 1998 292 S.

[108] Calvillo DP, Hawkins WC. Animate Objects are Detected More Frequently than Inanimate Objects in Inattentional Blindness Tasks Independently of Threat. The Journal of General Psychology. 2016;143(2):101–15. 10.1080/00221309.2016.1163249 27055078

[109] Vercayie D, Herremans M. Citizen science and smartphones take roadkill monitoring to the next level. Nature Conservation. 2015;11:29–40.

[110] Land-Zandstra AM, Devilee JLA, Snik F, Buurmeijer F, van den Broek JM. Citizen science on a smartphone: Participants’ motivations and learning. Public Understanding of Science. 2016;25(1):45–60. 10.1177/0963662515602406 26346340

[111] Shirk JL, Ballard HL, Wilderman CC, Phillips T, Wiggins A, Jordan R, u. a. Public participation in scientific research: a framework for deliberate design. Ecology and Society. 2012;17(2):1–29.

[112] Schlaepfer MA, Sax DF, Olden JD. The potential conservation value of non-native species: Conservation value of non-Native species. Conservation Biology. 2011;25(3):428–37. 10.1111/j.1523-1739.2010.01646.x 21342267

[113] Bradshaw CJA, Leroy B, Bellard C, Roiz D, Albert C, Fournier A, u. a. Massive yet grossly underestimated global costs of invasive insects. Nature Communications. 2016;7(1):1–8. 10.1038/ncomms12986 PMC5059451 27698460

[114] Kobori H, Dickinson JL, Washitani I, Sakurai R, Amano T, Komatsu N, u. a. Citizen science: a new approach to advance ecology, education, and conservation. Journal of Ecology Research. 2016;31:1–19.

[115] Deguines N, de Flores M, Loïs G, Julliard R, Fontaine C. Fostering close encounters of the entomological kind. Frontiers in Ecology and the Environment. 2018;16(4):202–3.

[116] Goulson D. Effects of introduced bees on native ecosystems. Annual Review of Ecology, Evolution, and Systematics. 2003;34(1):1–26.

[117] Aizen MA, Morales CL, Vázquez DP, Garibaldi LA, Sáez A, Harder LD. When mutualism goes bad: density-dependent impacts of introduced bees on plant reproduction. New Phytologist. 2014;204(2):322–8.

[118] Geslin B, Gachet S, Deschamps-Cottin M, Flacher F, Ignace B, Knoploch C, u. a. Bee hotels host a high abundance of exotic bees in an urban context. Acta Oecologica. 2020;105:103556.

[119] Laport RG, Minckley RL. Occupation of active Xylocopa virginica nests by the recently invasive Megachile sculpturalis in upstate New York. Journal of the Kansas Entomological Society. 2012;85(4):384–6.

[120] Roulston T, Malfi R. Aggressive eviction of the Eastern Carpenter Bee (Xylocopa virginica (Linnaeus)) from its nest by the Giant Resin Bee (Megachile sculpturalis Smith). Journal of the Kansas Entomological Society. 2012;85(4):387–8.

[121] Kelner-Pillaut S. Un Sphex americain introduit dans le Sud de la France, Sphex (Iso-dontia mexicana) Harrisi Fernald. L’Entomologiste. 1962;18:102–10.

