# Supplementary material for: Dispersal patterns of an introduced wild bee, Megachile sculpturalis Smith, 1853 (Hymenoptera: Megachilidae) in European alpine countries
Source: PLoS One. 2020 Jul 10;15(7):e0236042. doi: 10.1371/journal.pone.0236042 (PMC7351169; doi:10.1371/journal.pone.0236042)
Supplement: S1 Table — (PDF) [file pone.0236042.s001.pdf]

supporting institution	description	year of call	nation	website
Käfer&co	entomological club Bern	2018, 2019	CH	
entomological community Zurich	facebook discussion forum	2018, 2019	CH	https://www.facebook.com/groups/604731403215180
Wildbiene + Partner	company selling bee hotels based in Zurich	2019	CH	https://wildbieneundpartner.ch
nature&land	print media of the "Naturschutzbund Österreich"	2018	AUT	https://naturschutzbund.at
naturbeobachtung	online platform associated with the Naturschutzbund Österreich	2019	AUT	https://www.naturbeobachtung.at
inatura Erlebnis Naturschau GmbH	nature museum	2019	AUT	https://www.inatura.at
social media	instagram blog	2019	Int.	https://www.instagram.com
