# Supplementary material for: Dispersal patterns of an introduced wild bee, Megachile sculpturalis Smith, 1853 (Hymenoptera: Megachilidae) in European alpine countries
Source: PLoS One. 2020 Jul 10;15(7):e0236042. doi: 10.1371/journal.pone.0236042 (PMC7351169; doi:10.1371/journal.pone.0236042)
Supplement: S2 Table — (PDF) [file pone.0236042.s002.pdf]

country	zip code	location	individuals	observation type	year of observation
	6900	Bregenz	1	nest building in wooden furniture	2019
	6900	Bregenz	3	artificial nest	2019
	6900	Bregenz	1	nest building in roof beams	2019
	6850	Dornbirn	2	artificial nest	2019
	7000	Eisenstadt	1	plant interaction *Centaurea scabiosa*	2019
	6820	Frastanz	3	artificial nest	2019
	6673	Grän	1	plant interaction *Lavandula sp.*	2019
	8020	Graz	1	found dead	2019
	6992	Hirschegg	1	artificial nest	2019
	6973	Höchst	2	plant interaction *Vitus agnus-castus*	2019
	6845	Hohenems	1	nest building in roof beams	2019
	6914	Hohenweiler	1	artificial nest	2019
	6912	Hörbranz	1	plant interaction *Lathyrus latifolius*	2019
	9020	Klagenfurt	3	artificial nest	2019
Austria	6923	Lauterach	2	artificial nest	2019
	6923	Lauterach	2	artificial nest	2019
	6923	Lauterach	2	plant interaction *Eupatorium fistulosum*	2019
	6951	Lingenau	1	plant interaction *Lavandula sp.*	2019
	6890	Lustenau	3	artificial nest	2019
	6890	Lustenau	1	found dead	2019
	6891	Lustenau	3	artificial nest	2018, 2019
	6890	Lustenau	1	artificial nest	2019
	6890	Lustenau	1	artificial nest	2019
	6812	Meiningen	2	plant interaction *Wisteria sp.*	2019
	6241	Radfeld	1	artificial nest	2019
	5020	Salzburg	7	artificial nest	2019
	5071	Salzburg	3	plant interaction *Betonica officinalis*	2019
	6780	Schruns	1	entangeld in hair of a volunteer	2019
	6112	Wattens	3	artificial nest	2019
