# Supplementary material for: Dispersal patterns of an introduced wild bee, Megachile sculpturalis Smith, 1853 (Hymenoptera: Megachilidae) in European alpine countries
Source: PLoS One. 2020 Jul 10;15(7):e0236042. doi: 10.1371/journal.pone.0236042 (PMC7351169; doi:10.1371/journal.pone.0236042)
Supplement: S3 Table — Abbreviation: nd = no data of adult individuals available as only the nest was observed. (PDF) [file pone.0236042.s003.pdf]

country	zip code	location	individuals	observation type	year of observatons
	8910	Affoltern am Albis	1	artificial nest	2018
	8450	Andelfingen	6	artificial nest	2017
	3013	Bern	3	artificial nest	2017
	3027	Bern	nd	artificial nest	2018
	8122	Binz	1	artificial nest	2018
	1022	Chavannes-près-Renens	1	plant interaction *Lavandula sp.*	2018
	1263	Crassier	1	artificial nest	2017
	6020	Emmen	1	artificial nest	2018
	6376	Emmetten	3	artificial nest	2016
	5304	Endingen	1	artificial nest	2019
	8706	Feldmeilen	2	artificial nest	2019
	8500	Frauenfeld	2	artificial nest	2019
	8500	Frauenfeld	1	abandoned cavity of *Xylocopa sp.*	2018, 2019
	8500	Frauenfeld	2	artificial nest	2018
	7414	Fürstenau	5	artificial nest	2017
	1227	Genf	6	artificial nest	2018
	1265	Genf	1	artificial nest	2018
	6576	Gerra	1	artificial nest	2018, 2019
	8152	Glattpark	1	artificial nest	2017
	4465	Hemmiken	1	artificial nest	2018
	5502	Hunzenswil	3	artificial nest	2018
	3510	Konolfingen	4	artificial nest	2018
	8135	Langnau a.A.	4	artificial nest	2017, 2019
	3177	Laupen	2	artificial nest	2017
	1018	Lausanne	4	artificial nest	2018, 2019
	1007	Lausanne	2	artificial nest	2018, 2019
	6045	Meggen	1	artificial nest	2018, 2019
	8706	Meilen	3	artificial nest	2018
	8706	Meilen	3	artificial nest	2019
	6991	Neggio	2	found alive on soil	2019
	3176	Neuenegg	1	artificial nest	2018

	3653	Oberhofen am Thunersee	3	artificial nest		2018
Switzerland	8942	Oberrieden	5	artificial nest		2018, 2019
	8640	Rapperswil	1	artificial nest		2017, 2018, 2019
	8545	Rickenbach Sulz	2	artificial nest		2018, 2019
	9400	Rohrschach	5	artificial nest		2018, 2019
	6575	San Nazzaro	2	artificial nest		2019
	9430	Sankt Margarethen	4	artificial nest		2018, 2019
	9430	Sankt Margarethen	1	artificial nest		2018
	7320	Sargans	6	artificial nest		2018, 2019
	7320	Sargans	nd	artificial nest		2018
	8952	Schlieren	1	artificial nest		2018
	3960	Sierre	1	artificial nest		2018
	2072	St-Blaise	11	artificial nest		2018, 2019
	8712	Stäfa	14	artificial nest		2017
	3612	Steffisburg	3	artificial nest		2017
	8260	Stein am Rhein	1	artificial nest		2018
	1180	Tartegnin	1	artificial nest		2016
	7203	Trimmis	1	artificial nest		2017
	3614	Unterlangenegg	1	artificial nest		2019
	8918	Unterlunkhofen	1	artificial nest		2017
	3930	Visp	4	artificial nest		2018, 2019
	5430	Wettingen	1	artificial nest		2017
	8620	Wetzikon	1	artificial nest		2016
	6062	Wilen	1	artificial nest		2017, 2019
	8003	Zürich Alt-Wiedikon	1	artificial nest		2018
	8048	Zürich Altstetten	nd	artificial nest		2018
	8002	Zürich Enge	nd	artificial nest		2018
	8032	Zürich Hirslanden	2	artificial nest		2019
	8049	Zürich Höngg	1	plant interaction	*Lathyrus latifolius*	2019
	8049	Zürich Höngg	1	artificial nest		2019
	8006	Zürich Oberstrass	10	artificial nest		2017
	8050	Zürich Oerlikon	1	artificial nest		2019

	8057	Zürich Oerlikon	4	artificial nest	2018
	8052	Zürich Seebach	1	artificial nest	2018
Lichtenstein	9406	Balzers	1	artificial nest	2019
