# Supplementary material for: Dispersal patterns of an introduced wild bee, Megachile sculpturalis Smith, 1853 (Hymenoptera: Megachilidae) in European alpine countries
Source: PLoS One. 2020 Jul 10;15(7):e0236042. doi: 10.1371/journal.pone.0236042 (PMC7351169; doi:10.1371/journal.pone.0236042)
Supplement: S4 Table — (PDF) [file pone.0236042.s004.pdf]

country	year of obsrevation	location	source
Italy	2016	Meran	Guariento *et al.* 2019
	2019	Bruneck	Guariento *et al.* 2019
	2019	Meran	Guariento *et al.* 2019
	2019	Bruneck	Guariento *et al.* 2019
	2019	Bozen	Guariento *et al.* 2019
Germany	2015	Langenargen	Westrich *et al.* 2015
	2016	Überlingen	Westrich 2020
	2018	Insel Mainau	Westrich 2020
	2018, 2019	Freiburg	iNaturalist
	2019	Gschwend	Westrich 2020
	2019	Nonnweiler	Westrich 2020
	2019	Traunstein	Westrich 2020
	2019	Kapfing bei Vilsheim	Westrich 2020
	2019	Karlsruhe-Hagsfeld	Westrich 2020
	2019	Allensbach am Bodensee	Westrich 2020
	2019	Aalen-Unterkochen	Westrich 2020
	2019	Burgthann/Dörlbach	Westrich 2020
	2019	Stubenberg	Westrich 2020
	2019	Tuttlingen	Westrich 2020
	2019	Karlsruhe-Durlach	Westrich 2020
	2019	Rosenheim	Westrich 2020
	2019	Baierbach	Westrich 2020
	2019	München	Westrich 2020
Slovenia	2016	Trentavalley	Gogola & Zadravec 2018
	2018	Ljubljana	gbif
Switzerland	2010	Camedo	info fauna
	2012	Brissago	info fauna
	2012	Ronco	Amiet 2012
	2015	Zürich	Westrich *et al.* 2015
	2016	Martigny	info fauna
	2016	Altdorf	Dillier 2016
	2017, 2018	Petit-Lancy	info fauna
	2018	Minusio	Rickenbach & Sprecher 2018
	2018	Vernier	info fauna
	2018	Lausanne	info fauna
	2019	Saas-Grund	unpublished data Westrich 2019
	2019	Ardon	iNaturalist
	2019	Rebstein	iNaturalist
Austria	2017, 2018, 2019	Wien	Wiesbauer 2017
	2018	Mödling	iNaturalist
