# Supplementary figures and images for: Dispersal patterns of an introduced wild bee, Megachile sculpturalis Smith, 1853 (Hymenoptera: Megachilidae) in European alpine countries
Source: PLoS One. 2020 Jul 10;15(7):e0236042. doi: 10.1371/journal.pone.0236042 (PMC7351169; doi:10.1371/journal.pone.0236042)

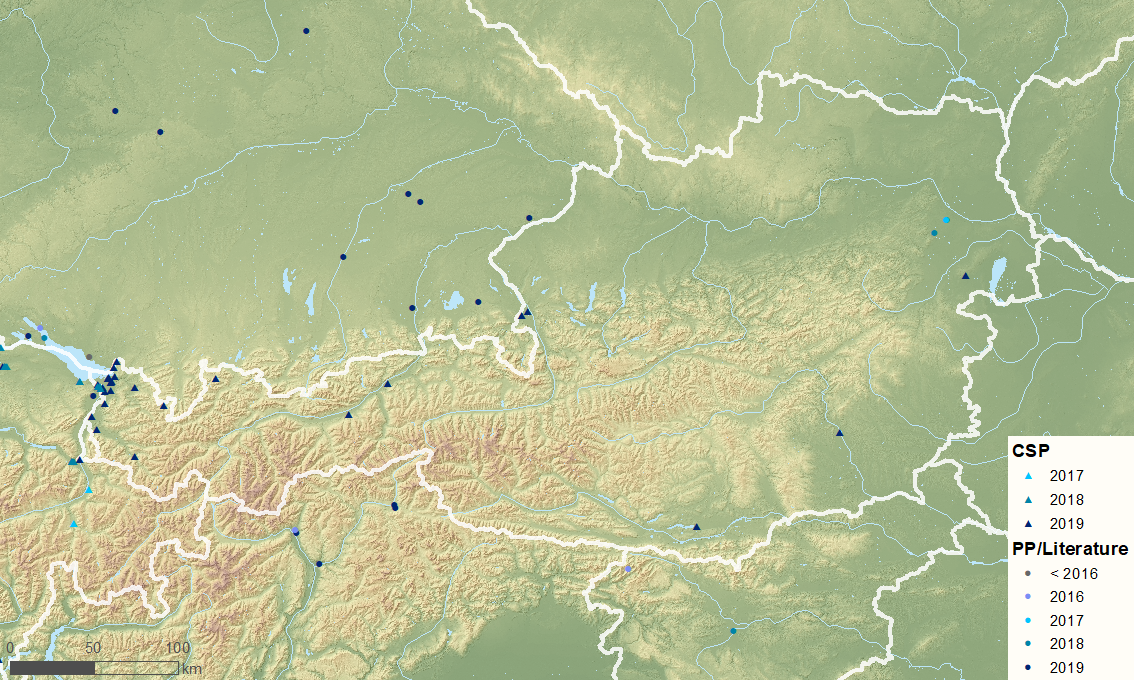

Supplement: S1 Fig — (PNG) [file pone.0236042.s007.png]

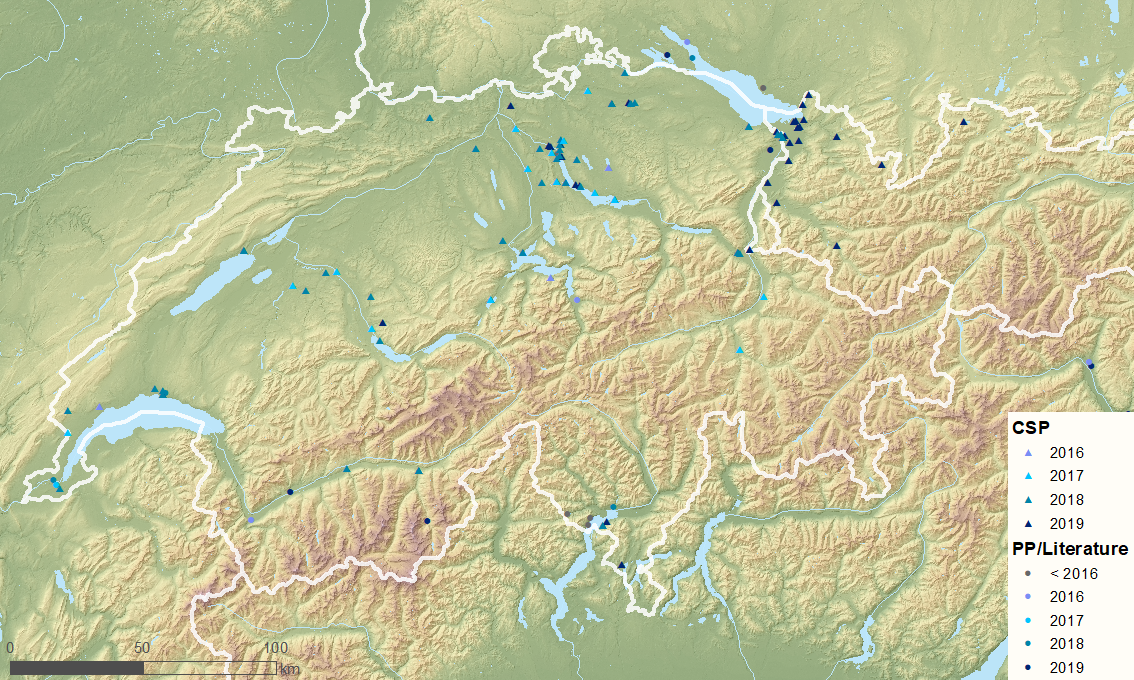

Supplement: S2 Fig — Abbreviation: nd = no data of adult individuals available as only the nest was observed. (PNG) [file pone.0236042.s008.png]
